# As a component of aseptic technique, Bunsen burners do not universally decrease deposition of microbe-containing particles

**DOI:** 10.1128/spectrum.01028-26

**Published:** 2026-08-17

**Authors:** Kate DeVincent, Ariana Carretta, Vanessa Colina, Imani Laporte, Tyler Oung, Hannah E. Gavin

**Affiliations:** 1 Department of Health Sciences, Providence College 736373, Providence, Rhode Island, USA; 2 Department of Biology, Providence College 6753, Providence, Rhode Island, USA; Duke University School of Medicine, Durham, North Carolina, USA; Duke University School of Medicine, Durham, North Carolina, USA

**Keywords:** asepsis, aseptic technique, sterile technique, Bunsen burner, airborne contamination, flame

## Abstract

Microbiologists often use Bunsen burners to reduce airborne microbial contamination in their workspace. In theory, the burner’s flame pushes air—and the microbe-containing particles therein—up and away from research materials. This is purported to create a disinfected zone that improves asepsis. While such use of a Bunsen burner is promoted in numerous textbooks, peer-reviewed publications, and university-affiliated websites, our search for data on Bunsen burner efficacy revealed a dearth of experimental evidence in scientific literature. Therefore, we designed and conducted a series of laboratory experiments to test the utility of this method, hypothesizing that the flame would reduce airborne contamination. We intentionally inoculated the air in a laboratory space with bacteria, then collected data on the deposition of airborne contaminants using a paired settle plate method. On average, plates adjacent to an active Bunsen burner do not experience a reduction in colony-forming units (CFU) settling. Linear regression indicates that results are environmentally context dependent: plates adjacent to Bunsen burners with 18 mm flame diameter exhibit mildly decreased deposition of dry particles when control contamination rates exceed 83 CFU/min, but increased dry particle deposition at contamination rates below that cutoff. Duration of flame activity is not associated with improved aseptic outcomes, regardless of flame size or particle moisture. Taken together, these data suggest that 18 mm Bunsen burners should be selectively implemented only in situations of “high and dry” airborne contamination. Outside this specific context, their activity is either neutral or detrimental to laboratory asepsis.

**IMPORTANCE:**

The ability to manipulate and control microbial presence in built environments is imperative to research and medicine. The lack of documented evidence relating Bunsen burner use and airborne contamination led us to conclude that this element of aseptic technique has until now been supported primarily by lore and tradition rather than experimental data. Given that laboratory open flame use has financial, temporal, and safety implications for research institutions across the globe, we sought to determine whether it is a worthwhile investment of resources. Ultimately, our data suggest that this practice is not evidence-based and that, except in a specific subset of circumstances, Bunsen burners can be removed from classroom and laboratory practice without compromising research integrity.

## INTRODUCTION

The Pasteur Institute defines aseptic technique as “the various measures to be taken to prevent invasion of live tissue or inert environments by exogenous microorganisms or viruses” (1). More colloquially, aseptic technique has been defined as “the key factor in doing microbiological things the right way” and is generally used to mean an environment “free from any microorganisms that are unwelcome at a particular place and time” (2). In research, the ability to prevent extraneous microbes from contaminating study samples is deemed “crucial for accurate and meaningful experimentation” (3).

**Fig 1:**
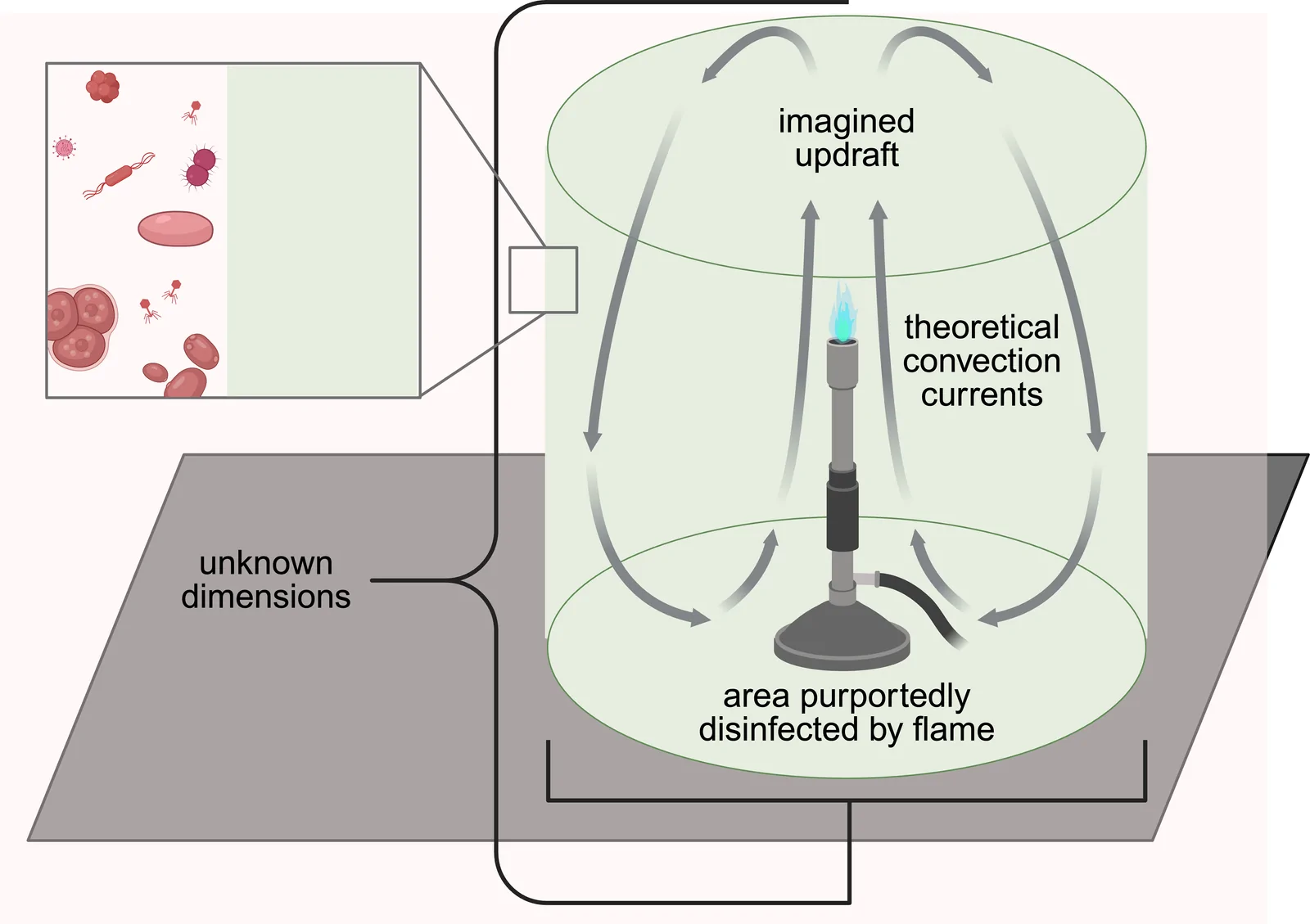
Several resources suggest that an active Bunsen burner flame produces convection currents, which in turn push contaminated air up and away from a work area proximal to the burner. Created in BioRender. Gavin, H. (2026) https://BioRender.com/euyapj5.

Aseptic technique encompasses a suite of behaviors and activities intended to minimize microbial contamination. Standard aseptic practices include sterilization of research materials, donning of personal protective equipment, and use of laminar flow cabinets. In addition, several published resources advocate for the application of Bunsen burners to reduce airborne contamination (3–8). Specifically, these resources imply that convection currents purportedly produced by the Bunsen flame carry airborne microbe-containing particles up and away from the materials being manipulated at a laboratory workstation (Fig. 1). Protocols instruct researchers to perform all work “close to the Bunsen burner flame where air currents are drawn upward” (9). Notably, however, the use of Bunsen burners to reduce airborne contamination is far from a uniformly applied practice. Several contemporary resources that implement Bunsen burners for sterilization of physical instruments (e.g., inoculating loops) make no claims about airborne decontamination. Moreover, the dimensions of the so-called “sterile field” generated by the burner are inconsistent even among sources that support its validity, and its reported radius ranges more than twofold (5, 8, 10).

As we analyzed literature relating Bunsen burners and asepsis, we realized that none of the resources espousing a burner-proximal aseptic zone cite experimental data. We are not the first to notice this knowledge gap: the lack of evidence was highlighted two decades ago by Melind Watve. He wrote in 1996 that “there is no authentic report that quantitatively studies the microbial load of the so-called aseptic zone” (11), and to our knowledge, this remains true in 2026. While we do not know of any formal analysis on current patterns and prevalence of Bunsen burner use among microbiologists, online forum chats and individual conversations with the corresponding author anecdotally reveal a range of attitudes regarding the relationship, if any, between Bunsen burners and asepsis (12–14). Some practitioners uphold the Bunsen burner as a critical component of asepsis, while their contemporaries claim it is completely unnecessary. Still others voice an intermediate view: that a flame is helpful only in very contaminated environments but dispensable in “clean” labs. A separate subset of comments suggests that a flame could make contamination worse, by the logic that the same convection currents drawing air upward and away from a flame may also pull microbe-containing air horizontally across a benchtop toward research samples (e.g. Fig. 1).

A void of empirical data has evidently allowed for the propagation of disparate and mutually exclusive views. We therefore designed a set of laboratory experiments to examine this practice. Given the logic behind convection-driven updrafts, we hypothesized that the Bunsen burner would positively impact aseptic technique. Specifically, we predicted that, compared to Petri plates adjacent to an inactive burner, plates proximal to an active burner would experience reduced deposition of airborne microbe-containing particles during equivalent exposure periods.

## RESULTS

To address our primary hypothesis, we designed and applied a paired settle plate method to quantify contaminants passively deposited on Petri dishes during a specified time frame. We simultaneously collected data for a control plate, adjacent to an inactive burner, and a paired test plate, adjacent to an active burner. Reasoning that the effect of the burner would likely be inversely correlated to distance from the base of the flame, we selected the minimum literature-cited distance (10 cm) for our experiments. We also predicted that flame size or moisture content of airborne particulate might impact outcomes. Therefore, we varied these parameters by intentionally distributing bacteria-containing “dry” particles in some trials and liquid bacterial suspension “wet” particles in other trials (see Materials and Methods for details). We also tested two Bunsen burner flame diameters by employing, in different trials, either standard classroom Bunsen burners of 18 mm diameter or larger-diameter burners of 39 mm. We quantified bacterial settling on paired plates by counting colony-forming units (CFU) of the intentionally inoculated bacterium.

We first wanted to test the prediction that an active Bunsen burner yields relative decreases in settled contaminants, across the range of tested bacterial inocula. Multiple analyses indicate that the CFU data we collected are lognormally distributed (Table S4). Therefore, we compared the control and test data from each condition using non-parametric Wilcoxon matched-pairs signed-rank tests. Relevant statistics for each condition are summarized in Table 1.

**TABLE 1:** Descriptive statistics and Wilcoxon matched pairs test

	Dry particulate		Wet particulate		Dry particulate	
	18 mm burner		18 mm burner		39 mm burner	
	Control	Test	Control	Test	Control	Test
Descriptive statistics						
Minimum	20	15	17	16	17	19
Maximum	213	221	275	315	382	367
Range	193	206	258	299	365	348
Median	89	73	43	49	58	54
95% CI of median	54, 96	62, 97	35, 56	38, 54	33, 115	20, 330
25th percentile	47	48	29	33	37	26
75th percentile	115	120	66	70	79	139
IQR	68	72	37	37	42	113
Wilcoxon matched-pairs						
Number of pairs	43		11		49	
Sum of signed ranks (W)	109		15		81	
*P* value	0.52		0.53		0.69	

**Fig 2:**
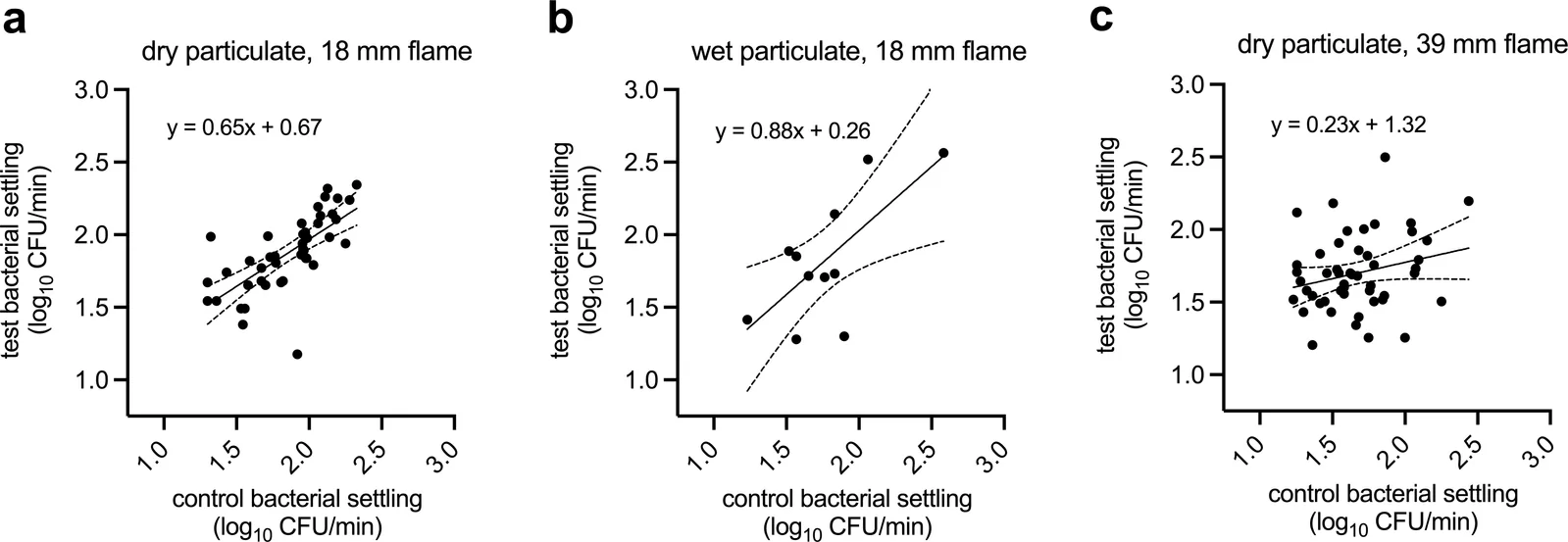
Log-transformed CFU that settled on open Petri plates under test (flame) conditions as a function of log-transformed CFU captured on control (no flame) plates. Linear regression yields the displayed equations. Dotted lines indicate the 95% CI: [0.44, 0.86] for panel a; [0.21, 1.56] for panel b; [−0.04, 0.49] for panel c. The r-squared values associated with each linear regression are (**a**) 0.48; (**b**) 0.50; (**c**) 0.06.

To test whether the effect of the Bunsen burner is dependent upon contamination level, we log-transformed the control and test CFU quantities and confirmed that the transformed data were normally distributed (Table S5). For each condition, we plotted test data on the *y*-axis as a function of control data on the *x*-axis and performed linear regressions (Fig. 2). The null hypothesis—that the effect of the Bunsen burner is not dependent on the number of microbe-containing particles in the surrounding atmosphere—would be supported by a linear regression with a slope indistinguishable from 1. Alternatively, slope values significantly different from 1 would support an alternative hypothesis that Bunsen burner impact is dependent upon the magnitude of airborne contamination.

**Fig 3:**
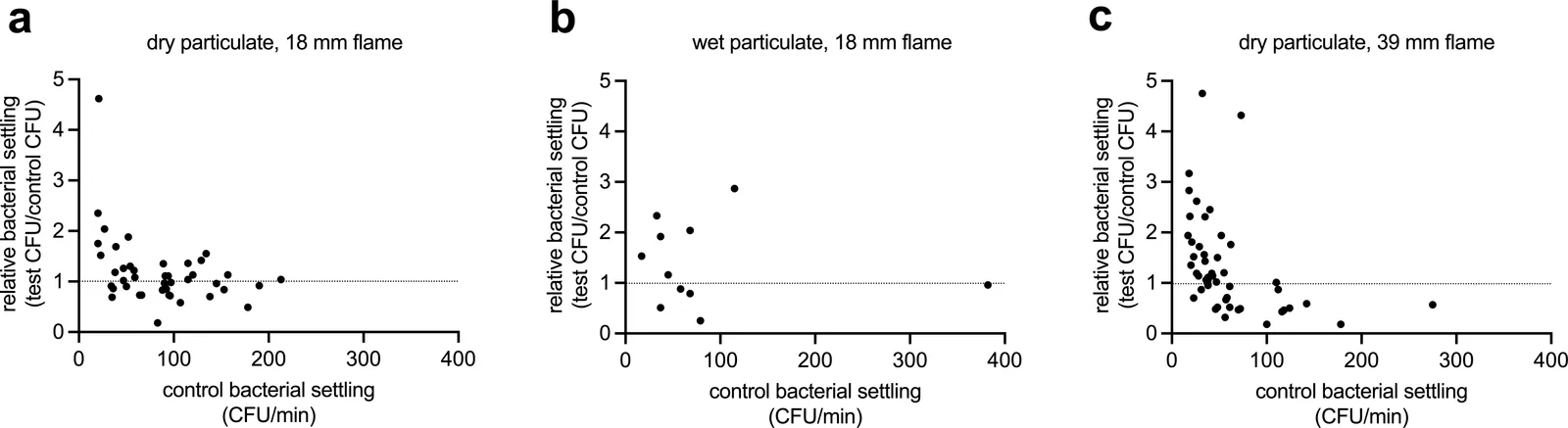
Relative number of CFU (test/control) as a function of contamination level. A relative settling value of 1 indicates equivalent CFU quantities on test and control plates; a value >1 indicates more CFU on test than control; a value <1 indicates less CFU on test compared to control. A visual reference line is plotted at *y* = 1. Data were collected under various contamination and flame conditions: dry microbe-containing particulates and a Bunsen burner flame with 18 mm diameter (**a**); wet microbe-containing particulates and a Bunsen burner flame with 18 mm diameter (**b**); and dry microbe-containing particulates and a Bunsen burner flame with 39 mm diameter (**c**).

We used the regression equations displayed in Fig. 2 to calculate the point at which Bunsen burner-adjacent microbe deposition is equivalent to control microbe deposition (i.e., where log_10_*y* = log_10_*x* and *y* = *x*). These values are *x* = 83 CFU/min for 18 mm flame/dry particles, 148 CFU/min for 18 mm flame/wet particles, and *x* = 52 CFU/min for 39 mm flame/dry particles, respectively. At *x*-values less than the indicated points, average test values exceed average control values. At *x*-values greater than the calculated equivalence points, CFU deposition on test plates is less, on average, than CFU deposition on control plates. As an alternative method to visualize the effect of inoculum dose on test CFU deposition, we calculated relative CFU values (test CFU/control CFU) for each data pair and plotted these as a function of contamination level (Fig. 3).

**Fig 4:**
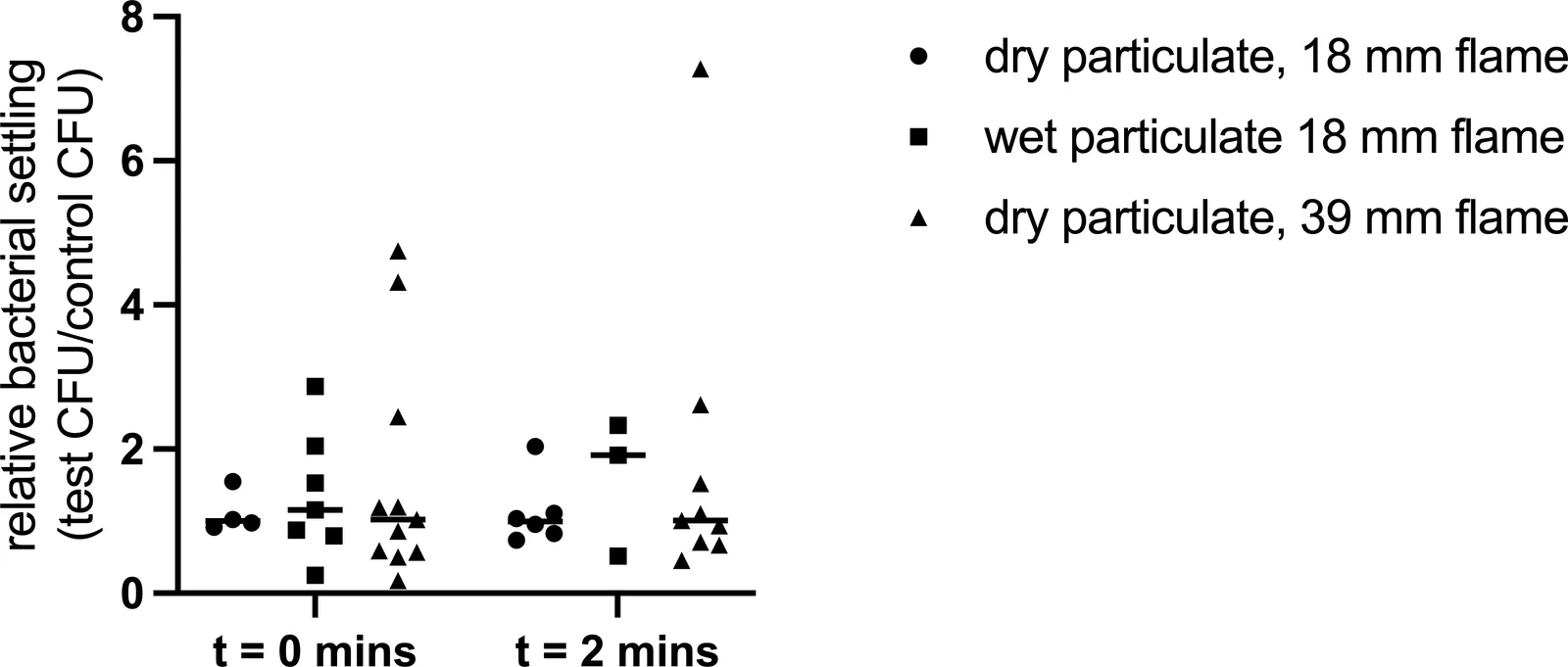
Relative settled CFU (test/control) as a function of time. Horizontal lines indicate median value. A Kruskal-Wallis test indicates no significant differences in median values between observed time points H (5, 40) = 0.342 and *P* = 0.997.

We next posited that a temporal delay in Bunsen burner efficacy would be observed if it takes some nonzero amount of time to establish the convection currents theorized in Fig. 1. We considered time as a variable and predicted that extended duration of flame activity would yield measurable improvement to asepsis. We compared CFU deposited on plates with zero minutes of prior flame activity to CFU deposited on flame-adjacent plates after two minutes of flame activity (Fig. 4). A Kruskal-Wallis test with multiple comparisons was performed and found no differences among any of the examined conditions.

## DISCUSSION

We first determine that the presence of an active Bunsen burner flame does not reduce average deposition of microbe-containing particles on test plates, compared to their control counterparts. For each set of conditions, the *P* value of a Wilcoxon matched-pairs signed-rank test far exceeds the typical *P* = 0.05 cutoff for statistically significant differences (Table 1). This initial analysis suggests that application of Bunsen burners has no net positive or negative effect and is effectively neutral in relation to asepsis. Notably, in this study we tested only one working distance (10 cm) from the base of the active Bunsen burner, and we cannot make any certain claims about relative contamination at locations more radially distant from the flame. However, given that any continuous sphere, cone, or cylinder with a larger radius (e.g., 20 cm) would inherently include the tested area, we can at least exclude the area with a 10 cm radius from the intimated “zone of sterility.”

Considering bacterial deposition on flame-proximal plates as a function of CFU on paired control (no flame) plates facilitates a more nuanced understanding of the observed results (Fig. 2 and 3). In experiments using an 18 mm Bunsen burner flame, control contamination level explains approximately 50% of the variation observed on test plates (*R*^2^ = 0.48 and 0.50 for dry and wet conditions, respectively). The null hypothesis is supported in the presence of wet particulates: a slope indistinguishable from 1 indicates that the flame confers no significant benefit or cost across the range of tested contamination levels. When airborne contamination consists of dry microbe-containing particles, however, the slope of the regression line differs significantly from 1 with 95% confidence (0.65 with 95% CI [0.44, 0.86]). Therefore, we observe differences in Bunsen burner utility dependent upon particle moisture. In the context of dry particulates, a slope significantly different from 1 leads us to reject the null hypothesis and consider alternatives.

Using the formula from the linear regression of the 18 mm dry particulate data (Fig. 2a), we calculated *x* = 83 CFU/min (95% CI [16, 6.23 × 10^4^]) to be the point at which burner-adjacent CFU deposition is equivalent to control CFU deposition (i.e., where log_10_*y* = log_10_*x* and *y* = *x*). In other words, Bunsen burner use is associated with an average increase in CFU deposition at contamination rates below the ~83 CFU/min cutoff. At settling rates >83 CFU/min, 18 mm flame activity is associated with decreased deposition of dry microbe-containing particulates (Fig. 2a and Fig. 3a). Therefore, we conclude that performing work 10 cm proximal to an 18 mm Bunsen burner flame is detrimental to asepsis where laboratory contamination, as measured by particle settling rate, falls below *x* = 83 CFU/min. Conversely, plates adjacent to an active burner experience mild benefits to asepsis when baseline settling rates exceed *x* = 83 CFU/min.

Together, these data support a conclusion that hybridizes several community-voiced predictions. The results at relatively high contamination levels are aligned with opinions about Bunsen burners being necessary only in “dirty” labs. The exacerbation of contamination observed at low CFU settling rates is consistent with the thought that burner-generated convection currents could actively pull contaminated air laterally across workbenches toward research materials. Ultimately, we conclude that 18 mm Bunsen burner impacts on asepsis are environmentally context-dependent. Benefits are restricted to dry particle contamination levels above 83 CFU/min; costs are associated with dry particle contamination levels below 83 CFU/min, and no net effect is observed in the presence of wet particle contamination.

For a Bunsen burner producing a 39 mm diameter flame, contamination magnitude explains only 6% of the observed variation in CFU deposition on burner-proximal plates (Fig. 2c). Therefore, other factors must have a more significant impact on asepsis (or lack thereof). In this study, we did not directly test or observe convection currents, but we posit that the 39 mm diameter Bunsen burner may generate more local turbulence relative to the 18 mm burner, which in turn leads to greater stochasticity. Relatedly, we were surprised to find that the duration of flame activity did not have an impact on settling rates for either burner size under any particle condition. Even with 2 min time to heat surrounding air and generate convection currents, there were no differences in relative contamination compared to control (Fig. 4). This suggests either that convection currents are immediately generated upon flame lighting, or that convection currents do not play as sizable or reliable a role in asepsis as has been previously conjectured. Future experiments could evaluate potential relationships among convection currents, turbulence, and CFU settling, potentially integrating electric Bunsen burners as a flame-free test condition.

Airborne microbes arise from a variety of sources including abiotic building materials, water (e.g., sinks and showers), cleaning practices (e.g., sweeping and vacuuming), animals, and the microbiomes of the human body itself (15–18). A 2015 study by Qian et al. found that human occupancy transiently increases airborne microbes up to 3.7 × 10^7^ bacterial genome copies per person-hour in a classroom setting, and that the majority of these were borne on large (supermicron) sized particles (19). However, neither deposition nor viability of shed microorganisms were evaluated, so it is difficult to directly relate these prior results to quantified settling rates from this study. We expect that differences in materials (e.g., carpet vs tile) and behavioral practices (e.g., use of PPE) significantly mitigate the impact of human occupancy on airborne microbial load in laboratories, compared to lecture classrooms, but this remains to be experimentally determined. Given that the “high” contamination rates tested in this study exceed un-inoculated conditions in our own lab space by several orders of magnitude—and could be achieved transiently for only minutes after intentional and aggressive airborne inoculation—we predict that the majority of teaching and research laboratory environments in the United States will typically fall below the calculated 83 CFU/min settle rate cutoff suggested by our work. Fortunately, microbe-containing particle deposition rates are easily testable; therefore, we recommend that a simple set of settle plate assays be conducted *in situ*. If a research environment regularly exhibits contaminant settling rates beyond 50–100 CFU/min, investigators can make a conscious decision to implement Bunsen burners in an appropriately data-driven manner.

Airborne particles exhibit size-dependent variations in aerial persistence and surface deposition, leading to a bimodal distribution in which small and large particles settle on surfaces more readily than mid-sized particles (20–22). Connecting these basic science observations to public health, the particle size variable directly relates to differences in pathogen transmissibility. While researchers have recently argued that past use of “droplet” versus “aerosol” categorization is a falsely simplified size binary, it remains accepted that physical differences in properties of pathogen-containing particles significantly influence host exposure and disease spread (20, 23). In this study, we did not control the diameter of distributed dry or wet particles. Instead, we aimed to dispense a heterogeneity of particle size and number using methods that mimic human-associated behaviors, such as clothing fabric rustling or a researcher sneezing. Based upon their visibility in the air and on the lab bench, we know that some distributed particulates fall within the “course” size category abundant in occupied built environments, but we did not test the size distribution of particles or quantify their relative abundance (19). Our chosen settle plate method for quantification is biased toward small and large (but not medium) particles with higher indoor deposition rates, particularly in the absence of direct airflow (22). Therefore, it remains possible for future studies to test questions about the influence of the Bunsen burner on particle suspension and size distribution through use of a laser counter or filter capture methods. We employed quantification of microbe-containing particle settling because it is arguably the most relevant metric to evaluate the contended relationship among Bunsen burners, airborne contaminants, and laboratory asepsis.

The results we present here echo the findings of a 1972 Applied Microbiology publication focused on another common aseptic practice: flaming the mouth of reusable test tubes. Authors Brunker and Fernandez were motivated by “contradictory indications concerning sterile tube inoculation […] in a number of textbooks” and noted that each of the various opposing techniques could be theoretically supported by logical arguments. The team designed and performed a set of experiments to examine this element of aseptic technique and ultimately concluded that flaming a culture tube “does not reduce contamination significantly and, hence, that this heretofore unquestioned ritual may represent only a waste of time.” Despite their well-designed study and conclusive findings, results from the Brunker and Fernandez study have been largely ignored: textbooks and laboratory manuals into the 2010s continued to espouse flaming test tube mouths as beneficial (3, 24). The 2020 edition of Current Protocols in Microbiology concedes in its Aseptic Technique chapter that “the value of ‘flaming’ has been questioned” and that “the practice of flaming is not necessary,” but then goes on to instruct that “if users choose to include flaming, then a brief […] pass of opened tubes through the burner flame should be added immediately after opening and just before closing containers” (4). The incongruity of these recommendations feels like an acknowledgment that what researchers know intellectually might conflict with what they feel about losing a facet of agency over experimental outcomes. In his 1996 commentary, Watve noted the instinct to continue a habitual practice rather than admit its futility as his peers lamented that “working [with] burners may be ineffective, but what can we do if we can’t afford laminar flow systems?” (11).

While biosafety cabinets are the leading evidence-based solution for the reduction of airborne contamination, it remains true that such laminar flow devices are not financially or physically viable in many situations. Since airborne contamination correlates with particle deposition, the implementation of air quality control measures improves experimental asepsis (25). Both quantity and diversity of airborne microbes in built environments increase with human traffic and activity level, so decreasing unnecessary movement around ongoing experiments is a good standard laboratory practice (18). Climate control and HVAC systems further “filter out some airborne microbes” (18). Additional freestanding HEPA filters—popularized across academic campuses during the COVID-19 pandemic—have costs on the order of 10- to 100-fold less than biosafety cabinets.

The microbiology community can also consider exploring creative and historically informed solutions in future experiments about aseptic technique and laboratory best practices. Prior to the advent of laminar flow, research suggested that experimental manipulations “can most successfully be done in dust-free rooms that have no air currents” (26–28). Today, some “dead air” type PCR cabinets offer a “circulation-free environment with UV light sterilization” that might be applicable to more generalized asepsis (29). At several thousand dollars, these generally cost less than a laminar flow hood. Even more financially accessible are still air boxes constructed using materials available at a hardware store. These are popular among the mycology community for amateur mushroom cultivation (30). The utility of so-called still air methods in asepsis remains to be tested in modern laboratory settings, but if proven efficacious, may provide a financial and logistical middle ground between Bunsen burners and biosafety cabinets.

The use of open flames necessitates multifaceted resources. Temporal investment in research is up to 25% higher when Bunsen burners are implemented in test tube flaming (31). Money is required for installation of gas lines, purchase of Bunsen burners, and continuous acquisition of consumable gas. Investment in infrastructure is greater for buildings that must meet higher risk-associated fire codes. Relatedly, there are very real safety concerns when flammable fluids, research trainees, and open flames are co-localized. This study illustrates that environmental context influences optimal aseptic technique. In a subset of labs where dry particle contamination is routinely detected at high levels, the investments and risks associated with Bunsen burner use are potentially justifiable. However, our data simultaneously indicate the futility of applying a Bunsen burner without *in situ* analysis: in most study-tested conditions, flame activity was neutral or counterproductive. Moving forward, we suggest that research time, money, and energy will be best spent testing and implementing evidence-based best practices for laboratory asepsis that do not rely on Bunsen burners to reduce the impact of airborne contaminants.

## MATERIALS AND METHODS

### Preparation of workspace

Upon entering the laboratory, lab coats were put on, hands were washed, and gloves and face masks were put on. The surface of the lab table was wiped down with Clorox Disinfecting Wipes (Home Depot Internet #100352706, Model #4460001656, Store SKU #104691) and left to air-dry completely. A Bunsen burner was placed in the center of each active workbench station, resulting in a separation distance of approximately 80 cm.

### Preparation of bacterial inoculum

*Gordonia rubripertincta* strain NRRL B-16540 was streaked from frozen glycerol stocks onto 100 mm Tryptic Soy Agar Petri plates and grown at 30°C for 2 days before being stored at room temperature. A liquid culture was prepared by using aseptic technique to transfer 75 mL of Tryptic Soy Broth (TSB) to a sterile bottle, then inoculating it with two to three colonies of *G. rubripertincta* from plates that were at least 3 days old and no older than 2 weeks. Liquid cultures were grown at 25°C–30°C with shaking in 250 mL baffled flasks at 250 rpm for 48–72 h, resulting in dense liquid cultures.

For dispersal of dry particulates, pre-sterilized shop towels (purchased from Home Depot: HDX Cotton Painter’s Rags Internet #202302489, Model #S-99264, Store SKU #434479, dimensions: 32 cm × 30 cm or HDX Terry Towels Internet #203191792, Model #T-99634-HDX, Store SKU #390814, dimensions: 35.6 cm × 43.2 cm) were immersed in the liquid culture, then gently wrung out. Each towel was left to dry on the benchtop overnight for use in the next day’s experiments. For dispersal of wet (droplet) particulates, bacterial cultures were diluted in sterile TSB (1:500–1:10,000) and transferred to spray bottles (purchased from Home Depot: Ecolab Heavy Duty Spray Bottle 32 oz, Internet #322222016, Model #53004560, Store SKU #1008462,394).

### Experiments and data collection

**Fig 5:**
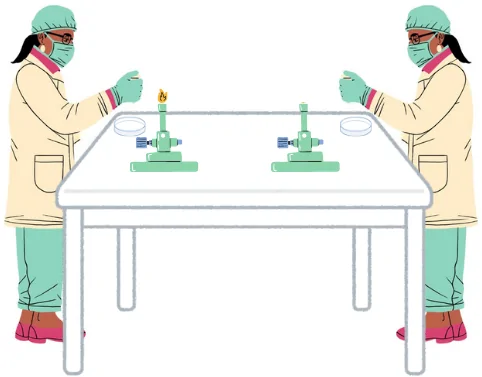
Schematic diagram of experimental setup. Researchers worked in pairs at a surface-disinfected laboratory bench to collect CFU deposition data on either a control plate (pictured right, no active flame) or test plate (pictured left, active flame). PPE, including lab coats, gloves, goggles, and shower caps, were employed to protect both researchers and research materials. Figure created using Canva.

Inspired by the settle plate (also known as settling plate, gravity plate, or passive monitoring) method described previously, we developed a protocol for collecting paired data under control conditions (no flame) and test conditions (active Bunsen burner flame) (11, 32–34). First, prepared plates of Tryptic Soy Agar in sterile 100 mm Petri dishes were allowed to come to room temperature. “Preparation of Workspace” was completed at a lab table with two mirrored workbench stations so that test (with flame) and control (without flame) data points were collected simultaneously. Strikers and labeled plates were placed adjacent to each workstation. Two researchers, each wearing standard PPE plus lab goggles, disposable face masks, and shower caps, collaborated to perform each trial. One researcher staffed each workbench station (Fig. 5). For each trial of dry particulate experiments, a microbe-laden towel (see section “Preparation of bacterial inoculum” above) was shaken vigorously above the entire lab table for one minute to disperse microbes in the air. The single researcher dispersing microbes slowly circled the entire lab bench and counted the number of times the towel was shaken on each side of the bench to approximate even distribution. For experiments with wet particulates, two researchers each used a spray bottle (with the nozzles turned 90° or 180° from closed, depending on the trial) to distribute a predetermined number of evenly spaced sprays (3–4, depending on the trial) over their side of the bench. This was temporally coordinated with the paired research partner at the opposite workstation.

At each station, a plate was placed with its center point 10 cm from the center of the Bunsen burner base. This setup was standardized across experiments through repeated use of a workstation template on plywood, covered in a clear plastic garbage bag. The template was sterilized with Clorox Disinfecting Wipes at the beginning of a new day’s experiments. Each “trial” was defined by a single inoculation event. Trials yielded multiple data pairs when a sufficient proportion of initial microbial inoculum remained airborne to allow CFU quantification during multiple minute-long collection periods.

**Fig 6:**
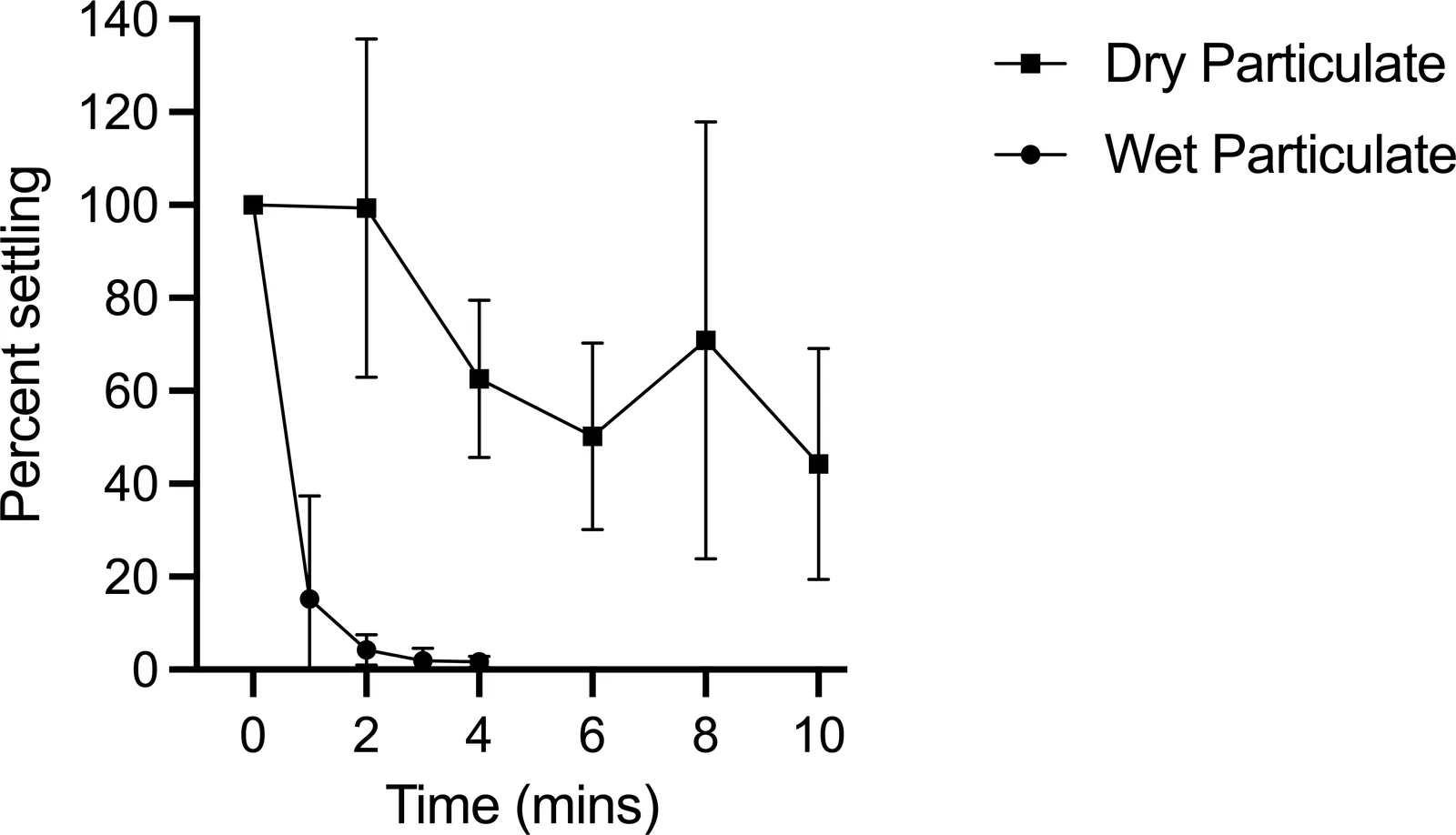
Temporal decay of airborne contaminants when microbes are inoculated into the air dry (from a shaken towel) or wet (from a spray bottle). Percents were calculated by dividing the CFU collected on plates at the indicated time by the CFU collected at time *T* = 0 (immediately following inoculation).

Over the course of several trials under each of the dry and wet conditions, we learned that the rate of decay of measurable dry particulates in the air was considerably slower than the observed rate of decay of wet particulates. Data from eight trials of each condition are displayed in Fig. 6. Following inoculation, there remained a quantifiable number of dry particulates in the air for a greater duration than the time of quantifiable wet particulates. Therefore, each trial of dry particulates yielded more paired data points compared to each trial of wet particulates and, relatedly, a greater number of individual trials with wet particulates ultimately yielded a smaller number of individual data points for quantification.

Burners were calibrated to generate a visible flame with a clear inner blue cone of approximately 4 inches and total flame height of approximately 6 inches. For most trials, the Bunsen burner on the “test” workbench was lit at time *T* = 0. Alternatively, for experiments assessing the impact of active flame duration on wet particulate settling, the flame was pre-lit for 2 min prior to airborne microbial inoculation and data collection. At designated time points, the Petri plate at each station was opened for 1 min, after which it was covered with its lid and set aside. This process was repeated at various intervals for the purpose of collecting data over a range of airborne microbial contamination magnitudes. Between different trials, the active Bunsen burner was switched to mitigate variables, such as room ventilation discrepancies. Depending on the experiment, one of two Bunsen burners was used: a standard burner with 18 mm diameter, or a larger burner with 39 mm diameter. While plates were open, researchers’ movement was minimized. Each individual sat at their station and moved in the theoretically decontaminated zone only when changing plates. All Petri plates were incubated at 30°C for 2 to 3 days before CFU quantification.

### Data analysis

For dry particulates, data were collected across nine trials with the 18 mm Bunsen burner and 11 trials with the 39 mm Bunsen burner. For wet particulates, data were collected across 11 trials with the 18 mm Bunsen burner. A paired data point was eliminated from analysis if any of the following conditions were met: either control or test plate CFU were <15; either control or test plate CFU exceeded 400; a non-*Gordonia* contaminant impeded quantification of the *G. rubripertincta* colonies on the plate (35). These criteria ultimately yielded, with the 18 mm burner, 43 paired data points for dry particulates and 11 paired data points for wet particulates. With the 39-mm diameter burner, 49 paired data points were analyzed.

GraphPad Prism Version 11.0.2 was used for data visualization and statistical analysis. For the data displayed in Fig. 2, linear regressions were performed on log-transformed data. Residual plots, QQ plots, and normality test results for each data set are available in Fig. S1. For the data displayed in Fig. 4, a non-parametric Kruskal-Wallis test with all possible group-by-group multiple comparisons was performed.

## Supplementary Material

Reviewer comments

## Data Availability

Raw CFU data are available in Tables S1 through S4.

## SUPPLEMENTAL MATERIAL

Figure S1 spectrum.01028-26-s0001.tiff Residual plots (graphs a, b, c) and QQ plots (graphs e, f, g) resulting from normality analysis of log_10_ transformed CFU data.

Table S1 spectrum.01028-26-s0002.csv CFU data collected in experiments using Bunsen burners with 18 mm diameter and distribution of dry bacterium-containing particulates.

Table S2 spectrum.01028-26-s0003.csv CFU data collected in experiments using Bunsen burners with 18 mm diameter and distribution of wet bacterium-containing particulates.

Table S3 spectrum.01028-26-s0004.csv CFU data collected in experiments using Bunsen burners with 39 mm diameter and distribution of dry bacterium-containing particulates.

Table S4 spectrum.01028-26-s0005.xlsx CFU data collected across all experiments, presented by date, trial, and post-inoculation time point. Data points included in the analysis are displayed in black; those that do not meet inclusion criteria are displayed in grey.

Table S5 spectrum.01028-26-s0006.xlsx Analysis of raw CFU deposition numbers indicates that control and test data are log-normally distributed. Analysis performed in GraphPad Prism.

Table S6 spectrum.01028-26-s0007.xlsx After log_10_ transformation, data are normally distributed and ready for linear regression analysis.

OPEN PEER REVIEW reviewer-comments.pdf An accounting of the reviewer comments and feedback.

ASM does not own the copyrights to Supplemental Material that may be linked to, or accessed through, an article. The authors have granted ASM a non-exclusive, world-wide license to publish the Supplemental Material files. Please contact the corresponding author directly for reuse.
