# Supplementary material for: As a component of aseptic technique, Bunsen burners do not universally decrease deposition of microbe-containing particles
Source: Microbiol Spectr. 2026 Aug 17;14(10):e01028-26. doi: 10.1128/spectrum.01028-26 (PMC13689453; doi:10.1128/spectrum.01028-26)
Supplement: Supplementary file 1 [file reviewer-comments.pdf]

AMERICAN SOCIETY FOR MICROBIOLOGY | Microbiology Spectrum

# As a component of aseptic technique, Bunsen burners do not decrease average deposition of microbe-containing particles

Kate DeVincent, Ariana Carretta, Vanessa Colina, Imani Laporte, Tyler Oung, and Hannah Gavin

*Corresponding Author(s): Hannah Gavin, Providence College School of Nursing and Health Sciences*



## Review Timeline:

| | |
|---|---|
| Submission Date: | March 24, 2026 |
| Editorial Decision: | April 30, 2026 |
| Revision Received: | June 24, 2026 |
| Accepted: | June 25, 2026 |

*Editor: Bobby Warren*

*Reviewer(s): Disclosure of reviewer identity is with reference to reviewer comments included in decision letter(s). The following individuals involved in review of your submission have agreed to reveal their identity: Nicholas A Turner (Reviewer #2)*

## Transaction Report:

(Note: With the exception of the correction of typographical or spelling errors that could be a source of ambiguity, letters and reports are not edited. The original formatting of letters and referee reports may not be reflected in this compilation.)

**DOI: https://doi.org/10.1128/spectrum.01028-26**

Re: Spectrum01028-26 (As a component of aseptic technique, Bunsen burners do not decrease deposition of microbe-containing particles)

Dear Dr. Hannah E. Gavin:

Thank you for the privilege of reviewing your work. Below you will find my comments, instructions from the Spectrum editorial office, and the reviewer comments.

Please return the manuscript within 60 days; if you cannot complete the modification within this time period, please contact me. If you do not wish to modify the manuscript and prefer to submit it to another journal, notify me immediately so that the manuscript may be formally withdrawn from consideration by Spectrum.

**Revision Guidelines**
To submit your modified manuscript, log into the submission site at https://spectrum.msubmit.net/cgi-bin/main.plex. Go to Author Tasks and click the appropriate manuscript title to begin. The information you entered when you first submitted the paper will be displayed; update this as necessary. Note the following requirements:

• Upload point-by-point responses to the issues raised by the reviewers in a file named "Response to Reviewers," NOT in your cover letter.
• Upload a compare copy of the manuscript (without figures) as a "Marked-Up Manuscript" file.
• Upload a clean .DOC/.DOCX version of the revised manuscript and remove the previous version.
• Each figure must be uploaded as a separate, editable, high-resolution file (TIFF or EPS preferred), and any multipanel figures must be assembled into one file.
• Any supplemental material intended for posting by ASM should be uploaded with their legends separate from the main manuscript. You can combine all supplemental material into one file (preferred) or split it into a maximum of 10 files with all associated legends included.

For complete guidelines on revision requirements, see our Submission and Review Process webpage. Submission of a paper that does not conform to guidelines may delay acceptance of your manuscript.

**Data availability:** ASM policy requires that data be available to the public upon online posting of the article, so please verify all links to sequence records, if present, and make sure that each number retrieves the full record of the data. If a new accession number is not linked or a link is broken, provide Spectrum production staff with the correct URL for the record. If the accession numbers for new data are not publicly accessible before the expected online posting of the article, publication may be delayed; please contact production staff (Spectrum@asmusa.org) immediately with the expected release date.

**Publication Fees:** For information on publication fees and which article types are subject to charges, visit our website. If your manuscript is accepted for publication and any fees apply, you will be contacted separately about payment during the production process; please follow the instructions in that e-mail. Arrangements for payment must be made before your article is published.

**ASM Membership:** Corresponding authors may join or renew ASM membership to obtain discounts on publication fees. Need to upgrade your membership level? Please contact Customer Service at Service@asmusa.org.

The ASM Journals program strives for constant improvement in our submission and publication process. Please tell us how we can improve your experience by taking this quick Author Survey.

Thank you for submitting your paper to Spectrum.

Sincerely,
Bobby Warren
Editor
Microbiology Spectrum

Reviewer #1 (Comments for the Author):

Major Concerns
Introduction
• Lines 129-137 - I believe the use of online forums should be more explicitly framed as anecdotal/contextual, not equivalent to scientific sources.

Discussion
• Lines 243-255 - the claim that burners may increase contamination in typical labs is important, but based on extrapolation from artificial conditions it should be clearly framed as hypothesis-generating, not definitive
• Lines 338-355 -statements such as "standard practice is unhelpful at best and harmful at worst" and "Bunsen burner could be a thing of the past" are overgeneralized based on data
• Lines 356-399 - the discussion of alternatives is valuable but moves somewhat beyond the direct scope of the data. Consider framing as future directions or practical considerations.
Methods
• Lines 453-456 - only a single distance (10 cm) was tested. Given variability in literature (10-20 cm), this limits interpretation of the "zone of sterility."

Minor Concerns

Typographical errors:
• "tenant"   "tenet" (Lines 49-54)
• "this this realization" (Line 119)
• Stray bracket "]" (Line 330)
Terminology:
• Clarify definitions of "dry" vs. "wet" particulates
• Ensure consistent use of "standard" vs "large" burner

Reviewer #2 (Comments for the Author):

Having trained at an institution which did not support the use of Bunsen burners to reduce airborne contamination, I was initially rather surprised at the purpose of this paper (I was not aware people were still doing this) - though the authors describe sufficient current variation in practice that it seems published evidence is needed.

Overall, I think the authors make a sufficient case that this topic is of interest - though for editorial purposes I think the paper could be substantially shortened:
-While some introduction is definitely necessary to explain the historical/practical context of the question, it spans nearly 8 pages and is more in the narrative style one might see in a review or textbook
-For a research journal, consider removing table 1 and substantially shortening/consolidating the intro

On a similar note, the discussion section is also quite a bit longer than the data presented likely warrant and also includes quite a bit of additional explanation/context and even some rather duplicative text (lines 338-355).
-Would similarly consider substantially shortening

I do have a few methodological questions:

1) While quite well described overall, the dispersal practices described in lines 418-423 seem rather prone to variability. E.g., if towels are wrung out differently or shaken differently, I wonder how variable particle generation is. It would seem like there would be engineered solutions to produce more consistent airborne inocula.
-While recognizing that their mirror/paired design should help mitigate this substantially, one might still wonder whether dispersal was even across the environment.
-Perhaps as a supplement, can the authors share any summary statistics on the inter-replicate/inter-trial variability in inocula with these methods?

2) For linear regression, assessment of a few key assumptions is usually necessary to assure validity but I don't see comment on these directly:
-Did the authors assess linearity approximation (e.g., with a QQ norm plot for example)?
-Did the authors assess for potential outliers or overly influential points? Some of the points in Figure 5 particularly B and C show some features of potentially influential outliers
-At least for statistical validity/optimal fitting, I'm not used to seeing fixation upon a forced intercept

3) The ratio of settling values seems to become a bit post-hoc/subjective as the authors set cutoffs above or below 50. Would probably avoid as this seems to become rather speculative and not clearly prespecified.

4) On use of Kruskal-Wallis testing (lines 206-209): It is odd to see a non-parametric test performed after providing summary data more suited to presumably normally distributed data (means+SD).
-If the data are not normally distributed would recommend reporting median/IQR instead
-Would report actual result of Kruskal-Wallis test (I see no summary stat or p value)

In the end, I am pleased that the findings support the teaching I had received - mainly not to use Bunsen burners to reduce airborne contamination in the lab - though admittedly I don't know many who do use this practice.

I'm not sure the question or the data included quite warrant the substantial length of the intro and discussion and wonder if this might be better received by most readers if significantly shortened perhaps even to a briefer format.

**Summary**

The authors evaluate whether Bunsen burners reduce airborne microbial contamination using settle plate experiments under controlled conditions. The authors demonstrate that standard burners (18 mm) do not meaningfully reduce contamination, while larger burners (39 mm) may reduce deposition only under high, artificially generated contamination levels. The study challenges a long-standing assumption in aseptic technique and suggests that Bunsen burner use for environmental decontamination is not evidence-based.

**Major Concerns**

Introduction

- Lines 129-137 – I believe the use of online forums should be more explicitly framed as anecdotal/contextual, not equivalent to scientific sources.

Discussion

- Lines 243-255 – the claim that burners may increase contamination in typical labs is important, but based on extrapolation from artificial conditions it should be clearly framed as hypothesis-generating, not definitive
- Lines 338-355 -statements such as “standard practice is unhelpful at best and harmful at worst” and “Bunsen burner could be a thing of the past” are overgeneralized based on data
- Lines 356-399 – the discussion of alternatives is valuable but moves somewhat beyond the direct scope of the data. Consider framing as future directions or practical considerations.

Methods

- Lines 453-456 – only a single distance (10 cm) was tested. Given variability in literature (10-20 cm), this limits interpretation of the “zone of sterility.”

**Minor Concerns**

Typographical errors:

- “tenant” → “tenet” (Lines 49-54)
- “this this realization” (Line 119)
- Stray bracket “]” (Line 330)

Terminology:

- Clarify definitions of “dry” vs. “wet” particulates
- Ensure consistent use of “standard” vs “large” burner

**Overall Assessment**

This is a thought-provoking and important study addressing a widely accepted but weakly evidenced practice. The experimental design is creative and the findings are impactful. However, limitations in ecological validity, measurement approach, and statistical interpretation, along with overstated conclusions, should be addressed to strengthen the manuscript.

We thank the reviewers for truly constructive comments that have made the study and its associated manuscript better in tangible, meaningful ways – particularly regarding data analysis. This is genuinely the most helpful reviewer commentary that the corresponding author has ever received, and she feels that it reflects the best of what peer review has the potential to be. We are grateful to you for sharing your time and energy!

Below you will find our responses, highlighted in yellow, to each reviewer comment.

**Reviewer #1 (Comments for the Author):**

Please note that the more substantial changes suggested by Reviewer 2 have in some cases resulted in the elimination of the text relevant to the changes suggested by reviewer #1. We will note below the corrections, if the original text remains, or that the particular line/word/section has been eliminated or significantly altered.

Major Concerns
Introduction
• Lines 129-137 - I believe the use of online forums should be more explicitly framed as anecdotal/contextual, not equivalent to scientific sources.

Updated the introduction lines 73-77 to read: "While we do not know of any formal analysis on current patterns and prevalence of Bunsen burner use among microbiologists, online forum chats and individual conversations with the corresponding author anecdotally reveal a range of attitudes regarding the relationship, if any, between Bunsen burners and asepsis"

Discussion
• Lines 243-255 - the claim that burners may increase contamination in typical labs is important, but based on extrapolation from artificial conditions it should be clearly framed as hypothesis-generating, not definitive

We have made several updates to the discussion section in response to this feedback, primarlily to frame this claim as a hypothesis based on extrapolation of the data, and also to encourage in-situ testing for context dependent application of the Bunsen burner. Lines 220-229 now read: "Given that the 'high' contamination rates tested in this study exceed un-inoculated conditions in our own lab space by several orders of magnitude – and could be achieved transiently for only minutes after intentional and aggressive airborne inoculation – we predict that the majority of teaching and research laboratory environments in the United States will typically fall below the calculated 83 CFU/min settle rate cutoff suggested by our work. Fortunately, microbe-containing particle deposition rates are easily testable; therefore, we recommend that a simple set of settle plate assays be conducted *in situ*. If a research environment regularly exhibits contaminant settling rates beyond 50-100 CFU/min, investigators can make a conscious decision to implement Bunsen burners in an appropriately data-driven manner.

• Lines 338-355 -statements such as "standard practice is unhelpful at best and harmful at worst" and "Bunsen burner could be a thing of the past" are overgeneralized based on data

These statements have been eliminated during revision. Lines 304-312 now read “This study illustrates that environmental context influences optimal aseptic technique. In a subset of labs where dry particle contamination is routinely detected at high levels, the investments and risks associated with Bunsen burner use are potentially justifiable. However, our data simultaneously indicate the futility of applying a Bunsen burner without *in situ* analysis: in most study-tested conditions, flame activity was neutral or counterproductive. Moving forward, we suggest that research time, money, and energy will be best spent testing and implementing evidence-based best practices for laboratory asepsis that do not rely on Bunsen burners to reduce the impact of airborne contaminants.”

• Lines 356-399 - the discussion of alternatives is valuable but moves somewhat beyond the direct scope of the data. Consider framing as future directions or practical considerations.

This section has been = shortened and reframed around practical considerations and future experimental directions.

Methods

• Lines 453-456 - only a single distance (10 cm) was tested. Given variability in literature (10-20 cm), this limits interpretation of the "zone of sterility."

Added several lines to the discussion (153-158) to read “Notably, in this study we tested only one working distance (10 cm) from the base of the active Bunsen burner, and we cannot make any certain claims about relative contamination at locations more radially distant from the flame. However, given that any continuous sphere, cone, or cylinder with a larger radius (e.g. 20 cm) would inherently include the tested area, we can at least exclude the area with 10-cm radius from the intimated ‘zone of sterility’”

Minor Concerns

Typographical errors:

• "tenant" ◊ "tenet" (Lines 49-54)

paragraph eliminated during revision

• "this this realization" (Line 119)

corrected

• Stray bracket "]" (Line 330) →

stray bracket eliminated

Terminology:

• Clarify definitions of "dry" vs. "wet" particulates

Added more explicit initial definition in lines 100-103, and directed readers to methods for further explanation.

• Ensure consistent use of "standard" vs "large" burner
Reduced the uses of ‘standard’ in associated with ‘burner’, and included explicit reference to the diameter each time; likewise reduced the instances of ‘large burner” in favor of clarifying 39 mm diameter.

Reviewer #2 (Comments for the Author):

Having trained at an institution which did not support the use of Bunsen burners to reduce airborne contamination, I was initially rather surprised at the purpose of this paper (I was not aware people were still doing this) - though the authors describe sufficient current variation in practice that it seems published evidence is needed.

Overall, I think the authors make a sufficient case that this topic is of interest - though for editorial purposes I think the paper could be substantially shortened:
-While some introduction is definitely necessary to explain the historical/practical context of the question, it spans nearly 8 pages and is more in the narrative style one might see in a review or textbook
-For a research journal, consider removing table 1 and substantially shortening/consolidating the intro

- On a similar note, the discussion section is also quite a bit longer than the data presented likely warrant and also includes quite a bit of additional explanation/context and even some rather duplicative text (lines 338-355).
-Would similarly consider substantially shortening

We have eliminated several pages of introductory text, including the prior table 1, and will revise that as a separate manuscript submission suited to a history-of-biology focused journal. We also substantially condensed the discussion section. Overall, we reduced the total document word count from 7478 words to 6139 words in hopes it becomes more approachable for the intended audience.

I do have a few methodological questions:

1) While quite well described overall, the dispersal practices described in lines 418-423 seem rather prone to variability. E.g., if towels are wrung out differently or shaken differently, I wonder how variable particle generation is. It would seem like there would be engineered solutions to produce more consistent airborne inocula.

This is absolutely true, but we do not view the variability of inocula as a negative aspect of the practice. Instead, it allowed us to test a range of control contamination ‘doses’ using relatively approachable, affordable methods that are arguably more applicable to real-world scenarios

than many engineered solutions. The inconsistent airborne inoculum is reflected in the range of control (no-flame) values.

-While recognizing that their mirror/paired design should help mitigate this substantially, one might still wonder whether dispersal was even across the environment.

While uneven environmental dispersal is absolutely possible, the paired design and multiple independent trials should minimize any disparities.

-Perhaps as a supplement, can the authors share any summary statistics on the inter-replicate/inter-trial variability in inocula with these methods?

Because the inocula were intentionally varied (by varying, for example, the dilution of culture in which the shop towels were immersed), we don't think that the summary statistics of these data would be meaningful or helpful to interpretation – the variability will inherently be high. To alleviate this concern, we have now included the data parsed by date, time, and trial.

2) For linear regression, assessment of a few key assumptions is usually necessary to assure validity but I don't see comment on these directly:
-Did the authors assess linearity approximation (e.g., with a QQ norm plot for example)?

When we performed normality and lognormality tests of the raw CFU data, all datasets favored a lognormal distribution over a normal distribution. It was an oversight not do to these tests in the first place, and we are grateful for this actionable feedback. Finding the data to be lognormally distributed, we first performed a Wilcoxon matched-paired signed-rank test since this non-parametric method does not assume normal distribution. We then log-transformed the data – these log-transformed datasets then each passed four separate normality tests – and checked the QQ plot and residual plot. (These are now included as a supplementary figure.)

-Did the authors assess for potential outliers or overly influential points? Some of the points in Figure 5 particularly B and C show some features of potentially influential outliers

To address this issue, we first evaluated and revised our data inclusion parameters and from "A paired data point was eliminated from analysis if any of the following conditions were met: **both** control and test quantified CFU were < 20; either the control or test CFU exceeded 400" (lines 473-475 original submission) to "A paired data point was eliminated from analysis if any of the following conditions were met: **either** control **or** test plate CFU were < 15; either control or test plate CFU exceeded 400." This decision was made based upon historic data validity/reproducibility experiments regarding 'countable' numbers of CFU (revised reference #35) and concern that any plates below ~15 CFU should not be included in the analysis. This change, combined with the log transformations of the data, reduced outliers as demonstrated on the residual and QQ plots in Supplemental Figure 1.

-At least for statistical validity/optimal fitting, I'm not used to seeing fixation upon a forced intercept

We initially forced the intercept grounded in the real-world understanding that if there are no microbes in the environment (x=0), they will not be generated by the presence of a flame (y=0). However, the data re-analysis in response to comments above have resulted in elimination of the graphs for which the forced intercept analysis was used, as we are now interpreting from linear regressions of log-transformed data in Figure 2. These analyses do not have forced intercepts.

3) The ratio of settling values seems to become a bit post-hoc/subjective as the authors set cutoffs above or below 50. Would probably avoid as this seems to become rather speculative and not clearly prespecified.

Good point – we were basing the 50 CFU cutoffs on visual interpretation of the data originally graphed in Figure 6, but it was not based on mathematical calculations. The updated data analysis has allowed us to calculate the x value (aka environmental contamination level) 'cutoff points' for each experimental condition (combination of burner size and dry vs. wet particulate) at which the activity of the burner tips from detrimental to beneficial. These are included in the results section lines 129-131 and their implications discussed in lines 168-179.

4) On use of Kruskal-Wallis testing (lines 206-209): It is odd to see a non-parametric test performed after providing summary data more suited to presumably normally distributed data (means+SD).

-If the data are not normally distributed would recommend reporting median/IQR instead

Updated according to this recommendation. Now reporting median and IQR for raw data, and switched to linear regression of log-transformed data since the raw data are better suited to lognormal patterns than normal distributions. See Table 1, Figure 2, and supplementary data.

-Would report actual result of Kruskal-Wallis test (I see no summary stat or p value)
Now included in Figure 4 legend in associated with the relevant graph.

In the end, I am pleased that the findings support the teaching I had received - mainly not to use Bunsen burners to reduce airborne contamination in the lab - though admittedly I don't know many who do use this practice.

We are curious about true prevalence of use as well, and plan to follow up on this question in the next few years by conducting survey research (not to be included in the revision of this manuscript). We have noted in the introduction and discussion that "We do not know of any formal analysis on current patterns and prevalence of Bunsen burner use among researchers. However, online forum chats and individual conversations with the corresponding author

anecdotally reveal a range of attitudes regarding the nature of the relationship, if any, between Bunsen burners and asepsis” (lines 73-77).

I'm not sure the question or the data included quite warrant the substantial length of the intro and discussion and wonder if this might be better received by most readers if significantly shortened perhaps even to a briefer format.

We have eliminated several pages of introductory text, including the prior table 1, and will revise that as a separate manuscript submission suited to a history-of-biology focused journal. We also substantially condensed the discussion section. Overall, we reduced the total document word count from 7478 words to 6139 words in hopes it becomes more approachable for the intended audience.

Re: Spectrum01028-26R1 (As a component of aseptic technique, Bunsen burners do not decrease average deposition of microbe-containing particles)

Dear Dr. Hannah E. Gavin:

Your manuscript has been accepted, and I am forwarding it to the ASM production staff for publication. Your paper will first be checked to make sure all elements meet the technical requirements. ASM staff will contact you if anything needs to be revised before copyediting and production can begin. Otherwise, you will be notified when your proofs are ready to be viewed.

**Data Availability:** ASM policy requires that data be available to the public upon online posting of the article, so please verify all links to sequence records, if present, and make sure that each number retrieves the full record of the data. If a new accession number is not linked or a link is broken, provide production staff with the correct URL for the record. If the accession numbers for new data are not publicly accessible before the expected online posting of the article, publication may be delayed; please contact ASM production staff immediately with the expected release date.

**Publication Fees:** For information on publication fees and which article types have charges, please visit our website. We have partnered with Copyright Clearance Center (CCC) to collect author charges. If fees apply to your paper, you will receive a message from no-reply@copyright.com with further instructions. For questions related to paying charges through RightsLink, please contact CCC at ASM_Support@copyright.com or toll free at +1-877-622-5543. CCC makes every attempt to respond to all emails within 24 hours.

**ASM Membership:** Corresponding authors may join or renew ASM membership to obtain discounts on publication fees. Need to upgrade your membership level? Please contact Customer Service at Service@asmusa.org.

**Embargo Policy:** A press release may be issued as soon as the manuscript is posted on the Spectrum Latest Articles webpage. The corresponding author will receive an email with the subject line "ASM Journals Author Services Notification" when the article is available online.

The ASM Journals program strives for constant improvement in our submission and publication process. Please tell us how we can improve your experience by taking this quick Author Survey.

Thank you for submitting your paper to Spectrum.

Sincerely,
Bobby Warren
Editor
Microbiology Spectrum
